# Impact of a Web-Based Decision Aid on Socioeconomically Disadvantaged Patients’ Engagement in Breast Surgery Decision-Making: Stepped-Wedge Clinical Trial (Alliance-A231701CD)

**DOI:** 10.1245/s10434-025-17452-0

**Published:** 2025-05-17

**Authors:** Jessica R. Schumacher, Bret M. Hanlon, David Zahrieh, Paul J. Rathouz, Jennifer L. Tucholka, Grace McKinney, Angelina D. Tan, Catherine R. Breuer, Lisa Bailey, Anna M. Higham, Julie S. Wecsler, Alicia H. Arnold, Anthony J. Froix, Scott Dull, Andrea M. Abbott, Stephanie G. Fine, Kandace P. McGuire, Anna S. Seydel, Patricia McNamara, Selina Chow, Heather B. Neuman

**Affiliations:** 1 University of Wisconsin School of Medicine and Public Health, Madison, WI USA; 2 Alliance Statistics and Data Management Center, Mayo Clinic, Rochester, MN USA; 3 University of Texas at Austin Dell Medical School, Austin, TX USA; 4 Bay Area Tumor Institute, Oakland, CA USA; 5 Carle Cancer Center, Urbana, IL USA; 6 Stroger Hospital of Cook County, Chicago, IL USA; 7 Augusta University Health/Medical College of Georgia, Augusta, GA USA; 8 University of Texas-Southwestern, Dallas, TX USA; 9 Montana Cancer Consortium NCORP, Billings Clinic, Billings, MT USA; 10 Medical University of South Carolina, Charleston, SC USA; 11 University of New Mexico, Albuquerque, NM USA; 12 Virginia Commonwealth University/Massey Cancer Center, Richmond, VA USA; 13 Marshfield Clinic, Marshfield, WI USA; 14 Alliance Protocol Operations Office, University of Chicago, Chicago, IL USA; 15 University of Wisconsin Carbone Cancer Center, Madison, WI USA

## Abstract

**Background:**

Decision aids (DAs) may increase engagement in decision-making by addressing barriers that disproportionately impact socioeconomically disadvantaged patients. The impact of a breast cancer surgery DA on increasing patient engagement in decision-making was tested in clinics serving a high proportion of socioeconomically disadvantaged patients.

**Methods:**

A stepped-wedge trial was conducted with 10 National Cancer Institute Community Oncology Research Program clinics (Alliance for Clinical Trials in Oncology, June 2019 to December 2021). The clinics were randomized to time of transition from usual care (UC) to delivery of a web-based DA. Patients with stages 0–3 breast cancer eligible for surgery provided consent before a surgical consultation. Engagement was measured by Patient’s Self-Efficacy in Patient-Physician Interactions (PEPPI-5, follow-up survey) and count of Active Patient Behaviors (audio-recorded consultation). Intervention effects were tested with linear mixed-effects models, accounting for surgeon and clinic-level clustering, time, and enrollment after COVID. Heterogeneity of treatment effect by socioeconomic disadvantage (using the Area Deprivation Index) was assessed with an interaction term.

**Results:**

The study enrolled 576 patients, and 44 % (136/309) of the patients reviewed the DA. No significant difference in engagement was observed between DA and UC for PEPPI-5 (− 0.8; 95 % CI, − 2.1–0.6; *p* = 0.260) or Active Patient Behaviors (2.5; 95 % CI, − 4.1–9.2; *p* = 0.456). No heterogeneity of treatment effect was observed. Socioeconomic disadvantage was associated with fewer Active Patient Behaviors (− 5.9; 95 % CI, − 0.6–− 1.2; *p* = 0.013).

**Conclusion:**

This trial conducted in clinics that serve diverse populations, observed no significant relationship between a web-based DA and patient engagement. Future analyses will explore DA implementation, characteristics of patients who reviewed the DA, and persistent barriers to engagement.

Shared decision-making (SDM) has the potential to improve the quality of breast cancer surgical care and reduce health disparities.^1–9^ Most women with a diagnosis of breast cancer are candidates for both mastectomy and breast conservation, and patients’ values and preferences should influence the surgical decision reached. However, prior work suggests that women who are socioeconomically disadvantaged have reduced understanding of options for treating their breast cancer,^10–13^ are less likely to recall discussing a choice of breast surgery with their surgeon,^14,15^ and participate less actively in decision-making.^16^ This gap contributes to observed disparities in surgical care, with disadvantaged women less likely to undergo breast conservation or receive post-mastectomy reconstruction.^17–22^

Findings show that SDM interventions (e.g., decision aids [DAs]) may increase patient engagement in decision-making by addressing known barriers to patient engagement that disproportionately impact socioeconomically disadvantaged patients.^2^ These barriers include lack of awareness about treatment choices, patients’ perceptions that their personal input is not valued, and doctor-patient power imbalances.^1^ Understanding whether DAs increase disadvantaged patients’ engagement in decision-making is a critical step toward reducing disparities in care. This clinical trial tested the impact of a breast cancer surgery DA on increasing patient engagement in decision-making in clinics serving a high proportion of socioeconomically disadvantaged patients.

## Methods

Details of Alliance for Clinical Trials in Oncology (Alliance) A231701CD have been previously published (ClinicalTrials.gov Identifier: NCT03766009).^23^

### Setting

We recruited 10 surgical clinics in the National Cancer Institute Community Oncology Research Program (NCORP) that care for a high proportion of socioeconomically disadvantaged patients.^24^ Clinics were selected to represent geographically diverse settings (Table 1).

**Table 1:** Characteristics of patient participants

	Usual care (*n* = 264) *n* (%)	Decision aid (*n* = 309) *n* (%)	Total (*n* = 573) *n* (%)
Median age: years (range)	59 (30–87)	61 (27–90)	60 (27–90)
Race			
White	169 (64)	207 (67)	376 (66)
Black	60 (23)	62 (20)	122 (21)
Other	35 (13)	40 (13)	75 (13)
Hispanic or Latino	6 (2)	21 (7)	27 (5)
Median ADI decile number (range)	6 (1–10)	4 (1–10)	5 (1–10)
Socioeconomic disadvantage	73 (28)	59 (19)	132 (23)
T stage			
T0	40 (15)	54 (18)	94 (16)
T1/T2	188 (71)	223 (72)	411 (72)
T3/T4	20 (8)	29 (9)	49 (9)
Tx or missing	16 (6)	3 (1)	19 (3)
Nodal status			
N0	191 (72)	247 (80)	438 (76)
N1/N2/N3	38 (14)	46 (15)	84 (15)
Nx or missing	35 (13)	16 (5)	51 (9)
Enrollment post-COVID	98 (37)	281 (91)	379 (66)
Sites			
Augusta University Medical Center	20 (8)	10 (3)	30 (5)
Bay Area Breast Surgeons, Inc	19 (7)	10 (3)	29 (5)
Billings Clinic Cancer Center	18 (7)	2 (1)	20 (4)
Carle Cancer Center	18 (7)	65 (21)	83 (15)
John H. Stroger Jr Hospital of Cook County	15 (6)	22 (7)	37 (7)
Kapiolani Medical Center for Women and Children	26 (9)	39 (13)	65 (11)
Marshfield Medical Center	57 (22)	4 (1)	61 (11)
Medical University of South Carolina	27 (10)	70 (23)	97 (17)
University of New Mexico Cancer Center	8 (3)	57 (18)	65 (11)
Virginia Commonwealth University/Massey Cancer Center	56 (21)	30 (10)	86 (15)

*ADI* Area deprivation index

### Overall Trial Design

We conducted a stepped-wedge trial with seven waves (July 2019 to December 2021). All the clinics began the trial in the usual care (UC) arm and were randomized to the time of transition from UC to delivery of a web-based DA. Clinic-level randomization was stratified based on whether the site was a minority/underserved NCORP.

The Alliance for Clinical Trials in Oncology, the research base for this trial, was responsible for protocol development, data collection and management, statistical analysis, and overall study operations.^25^ The study was approved by the National Cancer Institute (NCI) Central Institutional Review Board and was monitored by the Alliance Data and Safety Monitoring Board. Data quality was ensured by review of data by the Alliance Statistics and Data Management Center and by the study chairperson following Alliance policies. 

### Intervention

We provided the patients in the DA arm with a web-based DA.^26,27^ Based on feedback from patient, nurse, and surgeon stakeholders, we delivered the DA before the surgical consultation because this timing was perceived to best match patients’ informational needs and prepare them to participate actively in the consultation. This is consistent with the literature.^28,29^ Implementation of the DA into the clinical work flow followed pilot work and included clinic site visits by the research team.^30,31^ After each clinic crossed over to the intervention arm, all new breast cancer patients receiving care within that clinic were offered and e-mailed a link to the web-based DA before the surgical consultation as a component of UC.

### Participants and Recruitment

Patients at the participating clinics were eligible if they were female, were 18 years or age or older, had newly diagnosed breast cancer (stage 0, 2, or 3), and were planning surgery. Patients were excluded if they had impaired decision-making capacity (e.g., dementia) or required an interpreter. The research teams at each clinic pre-screened clinic schedules. Patients were approached by a research coordinator before the surgical consultation, and informed consent was obtained.

### Data Collection

The patients in both the UC and DA arms of the study completed a survey at the time of consent, had their surgical consultation audio-recorded (and then transcribed), and completed a survey after the consultation visit (median, 6 days; range, 1–81 days). Data were abstracted from the electronic medical record. The patients received an incentive of $10 each for participating in the audio-recording and completing the follow-up survey.

### Measures

The primary outcome was patient engagement, which we measured in two ways. First, we collected Patient’s Self-Efficacy in Patient-Physician Interactions (PEPPI-5) on the follow-up survey after the surgical consultation.^32,33^ The PEPPI-5 is a total score ranging from 5 to 25, with higher scores indicating increased engagement.

Second, we measured Active Patient Behaviors, coded from the audio-recorded consultations.^34–36^ This was a summary count of a patient’s communicative behaviors during the interaction, with higher scores indicating increased Active Patient Behaviors. Subscale scores included counts of Asking Questions (patient makes utterance in interrogative form that asks for information or clarification), Expressions of Concern (patient expresses worry, anxiety, fear, frustration, etc.), and Assertive Responses (patient expresses her interests, perspective, and desires as in offering an opinion, stating preferences, making decisions or a request).

We assessed socioeconomic disadvantage using the Area Deprivation Index (ADI) measured at the zip+4 level (2020 version) and dichotomized at the eighth decile as disadvantaged (yes/no).^37,38^ We asked the patients in the DA arm whether they reviewed the DA before coming to the consultation and the patients in the UC arm whether they reviewed any information before coming to the consultation. We asked the patients in the DA arm about perceived helpfulness of the DA (scale 0–10, with 10 as “very helpful”).

### Analysis Plan

Analyses were intention-to-treat without consideration whether patients in the DA arm received or reviewed the DA. Descriptive analyses assessed between-arm differences in mean PEPPI-5 and Active Patient Behaviors using analysis of variance (ANOVA). Intervention effects for each of the primary outcomes were tested with linear mixed-effects models, accounting for surgeon and clinic-level clustering. Models were re-estimated with each of the Active Patient Behaviors subscores as outcomes. Intraclass correlations were calculated for surgeon and clinic for each primary outcome. Patients were included in the analytic cohort for the PEPPI-5 outcome if they had completed the follow-up survey. Patients were included in the analytic cohort for the Active Patient Behaviors outcome if they had evaluable audio recordings.

Stepped-wedge studies are one-directional crossover designs, and as such, analysis plans require a model for time effects. In our study, this was complicated because accrual to the trial was closed in March 2020 due to the COVID-19 pandemic. Sites resumed enrollment at varying times based on local circumstances.

To acknowledge and account for time trends, we included additional parameters in the model (wave and wave-squared) to flexibly control for time effects and to allow for a non-linear relationship between time and outcome measures. In addition, we included two parameters to account specifically for COVID-19 (an indicator for whether the patient enrolled before versus after March 2020 and the wave number when the clinic resumed enrollment).

We assessed for heterogeneity of treatment effect by socioeconomic disadvantage with an interaction term included in the main models. We performed sensitivity analyses, which included five additional patients who had been enrolled slightly outside the wave schedule and used multiple imputation to impute outcome measures for patients who were missing either PEPPI-5 (*n* = 63) or Active Patient Behaviors (*n* = 9) outcomes.^39^ We examined the correlation of auxiliary variables for both outcomes. Because none of the patient characteristics met the requirement for correlation of 0.4 to be included in the model, we decided to use age, race, and ADI decile number as auxiliary variables to impute missing outcome measures. We performed exploratory analyses to assess how enrollment after COVID impacted Active Patient Behaviors. We also conducted a treatment-compliant analysis including only patients in the DA arm who reported reviewing the DA before the consultation or patients in the UC arm who reported reviewing any information before the consultation (collected on the survey at the time of consent).

We estimated power based on a sample size of 563 patients and 23 surgeons. This represented a reduction from the original estimated sample size of 1050 patients. This reduction was largely due to the COVID-19 pandemic, which resulted in temporary study closures and changes to study procedures that contributed to slower than expected accrual. With this sample size, we estimated that we had 80 % power to detect effects as small as 2.5 and 90 % power to detect effects as small as 2.7 on PEPPI-5. For Active Patient Behaviors, we estimated that we had 80 % power to detect effects as small as 11.6 and 90 % power to detect effects as small as 13.2 points.

## Results

**Fig. 1:**
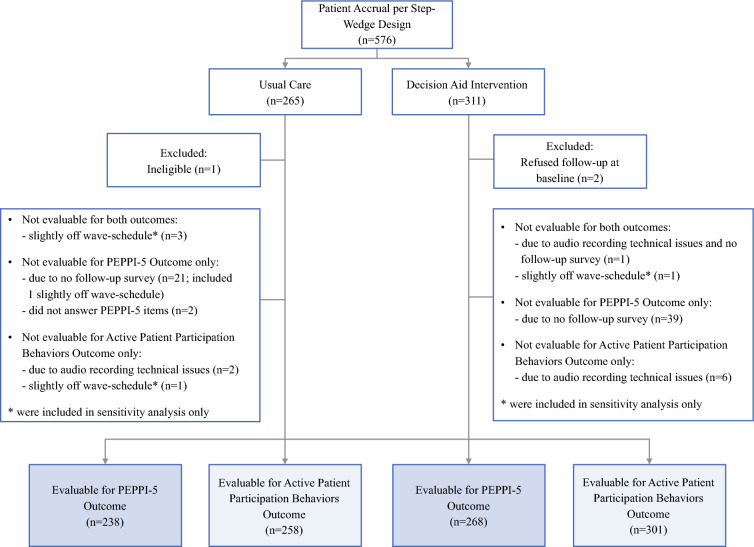
Overview of patient enrollment

Of the 576 patients who provided consent, 3 were excluded (final sample of 573; Fig. 1). The analysis for PEPPI-5 included 506 patients and the analysis for Active Patient Behaviors included 559 patients. The median patient age was 60 years (range, 27–90 years), and most of the patients had small, node-negative cancers. The cohort was racially diverse (66 % white; 21 % black). Based on the Area Deprivation Index, 23 % of the patients lived in an area of socioeconomic disadvantage (Table 1).

Overall, a minority of patients in the DA intervention arm (44 %) reviewed the DA before the consultation. Of the 309 patients in the DA arm, 198 (64 %) said they were offered the DA by the clinic. The majority of the patients offered the DA (94 %, 186/198) agreed to have it sent to them. Of the 186 patients who were sent the DA, 136 (73 %, 136/186) reviewed it before the consultation. The patients offered the DA by the clinic did not differ in terms of race, education, or socioeconomic disadvantage. Of the patients who agreed to be sent the DA, a lower proportion who were black (50 % versus 77 % white), lived in an area of high socioeconomic disadvantage (22 % versus 50 % living in an area of high disadvantage) or had a lower education (59 % with high school or less versus 89 % with graduate school degree) reviewed the DA. Most of the patients who reviewed the DA found it helpful (median score, 9/10; interquartile range [IQR], 7–10), and 93 % would have recommended it to other patients.

### Patient’s Self-Efficacy in Patient-Physician Interactions (PEPPI-5)

The mean PEPPI-5 score was 21.4 ± 3.4, with an observed ceiling effect (median score, 22.0; IQR, 20.0–24.0). There was no statistically significant difference in engagement as measured by the PEPPI-5 (Table 2a) between the DA and UC arms (− 0.8; 95 % CI, − 2.1 to 0.6; *p* = 0.260). In the heterogeneity of treatment-effect analysis, the effect of the DA on PEPPI-5 did not differ based on socioeconomic disadvantage (Table 2b).

**Table 2:** Impact of a web-based decision aid on engagement as measured by Patient’s Self-Efficacy in Patient-Physician Interactions (PEPPI-5) (*n* = 507)

Effect	Parameter estimate (95 % CI)	*p* Value
**2a** Main effect for the impact of a web-based decision aid on PEPPI-5^a^		
Decision aid versus usual care (reference)	− 0.8 (− 2.1 to 0.6)	0.260
Enrollment post-COVID: 1 versus 0 (reference)	− 1.3 (− 2.9 to 0.2)	0.096
Within-surgeon ICC = 0.0045 and within-site-between surgeon ICC = 0.0355.		

**2b** Heterogeneity of treatment effect by socioeconomic disadvantage for the impact of a decision aid on PEPPI-5^a^		
Decision aid versus usual care (reference)	− 0.5 (− 1.9 to 0.8)	0.443
Enrollment post-COVID: 1 versus 0 (reference)	− 1.4 (− 3.0 to 0.2)	0.082
Socioeconomic disadvantaged: 1 versus 0 (reference)	0.5 (− 0.5 to 1.4)	0.361
Decision aid Socioeconomic disadvantage^a^	− 0.9 (− 2.3 to 0.5)	0.183
Within-surgeon ICC = 0.0060 and within-site-between surgeon ICC = 0.0375		

*CI* confidence interval, *ICC* intraclass correlation coefficient

^a^Models adjusted for wave, wave-squared, and post-COVID restart time

### Active Patient Behaviors

The mean count of Active Patient Behaviors was 22.9 ± 18.1. The behavior observed most frequently was Asking Questions (Table 3), with Expressions of Concern uncommon. In a univariate analysis, a statistically significant difference was observed in the count of Assertive Responses between the DA and UC arms (8.4 versus 6.5; *p* = 0.006).

**Table 3:** Count of active patient behaviors overall and by study arm

	Usual care (mean ± SD)	Decision aid (mean ± SD)	Total (mean ± SD)
Total count	22.0 ± 17.8	23.7 ± 18.4	22.9 ± 18.1
Asking questions	14.8 ± 14.4	14.8 ± 13.4	14.8 ± 13.9
Assertive responses	6.5 ± 6.3	8.4 ± 7.8	7.5 ± 7.2
Expressions of concern	0.7 ± 1.6	0.5 ± 1.0	0.6 ± 1.3

*SD* standard deviation

The linear mixed-effects model showed no statistically significant difference in engagement as measured by Active Patient Behaviors (Table 4a) between the DA and UC arms (2.5; CI, − 4.1–9.2; *p* = 0.456). The heterogeneity of treatment-effect analysis showed no statistically significant difference in the impact of the DA on Active Patient Behaviors based on socioeconomic disadvantage (Table 4b). However, socioeconomic disadvantage was associated with significantly lower levels of engagement.

**Table 4:** Impact of a web-based decision aid on engagement as measured by active patient behaviors (*n* = 559)

Effect	Parameter estimate (95 % CI)	*p* Value
**4a** Main effect for the impact of a web-based decision aid on active patient behaviors		
Decision aid versus usual care (reference)	2.5 (− 4.1 to 9.2)	0.456
Enrollment post-COVID: 1 versus 0 (reference)	9.6 (1.8 to 17.4)	0.017
Within-surgeon ICC = 0.1082 and within-site-between surgeon ICC = 0.0461		

**4b** Heterogeneity of treatment effect by socioeconomic disadvantage for the impact of a decision aid on active patient behaviors^a^		
Decision aid versus usual care (reference)	2.2 (− 4.6 to 9.0)	0.525
Enrollment post-COVID: 1 versus 0 (reference)	9.5 (1.7 to 17.2)	0.017
Socioeconomic disadvantaged (yes versus no [reference])	− 5.9 (− 10.6 to − 1.2)	0.013
Decision aid socioeconomic disadvantage^a^	**− 0.2 (− 7.0 to 6.7)**	**0.962**
Within-surgeon ICC = 0.1127 and within-site-between surgeon ICC = 0.0415		

*CI* confidence interval, *ICC* intraclass correlation coefficient

^a^Models adjusted for wave, wave-squared, and post-COVID restart time

### Sensitivity Analyses

In our sensitivity analyses, no changes in the pattern of findings were observed (Appendix).

### Exploratory Analyses

Our treatment-compliant cohort included 136 patients in the DA arm and 154 patients in the UC arm. The treatment-compliant analysis was consistent with the primary analysis, with no statistically significant difference in engagement between the DA and UC arms (Appendix).

We observed that patient enrollment after COVID was associated with more Active Patient Behaviors (Table 4). In unadjusted and adjusted analyses, enrollment after COVID was associated with an increase in Asking Questions and Assertive Responses (Table 5).

**Table 5:** Impact of a web-based decision aid on subcomponents of active patient behaviors^a^

Effect	Total active patient behaviors	Parameter estimate (95 % CI)		
		Asking questions	Assertive responses	Expressions of concern
Decision aid versus usual care (ref)	2.5 (− 4.1 to 9.2)	0.9 (− 4.2 to 6.1)	1.8 (− 0.8 to 4.4)	− 0.03 (− 0.5 to 0.5)
Enrollment post-COVID: 1 versus 0 (ref)	9.6 (1.8 to 17.4)	6.8 (0.7 to 12.8)	3.1 (0.04 to 6.2)	0.02 (− 0.6 to 0.6)

*CI* confidence interval, *ICC* intraclass correlation coefficient

^a^Models included wave, wave-squared, enrollment post-COVID (yes/no), post-COVID restart time (in units of wave), within-surgeon ICC, and within-site-between surgeon

## Discussion

In this stepped-wedge clinic trial conducted within NCORP, we did not observe an effect of a web-based breast cancer surgery DA on patient engagement. We were able to enroll a diverse cohort of patients across our 10 participating clinics, with 35 % of the participants non-white and 23 % socioeconomically disadvantaged. The patients living in socioeconomically disadvantaged neighborhoods were less engaged in the consultation. However, the DA was not differentially effective based on socioeconomic disadvantage.

In this trial, we had low uptake of the DA, with only 44 % of the patients in the DA arm reviewing the DA before the consultation.^30,31^ This was markedly lower than the greater than 80 % uptake we observed in our pilot study.^30,31^ The lower than expected uptake can be partially explained by relatively low rates of patients offered the DA by clinics (64 %). We designed our trial to be pragmatic to account for known challenges to implementing DAs into clinical practice.^49^ Our implementation emphasized routine integration of the DA into clinical practice during the study period for all the patients irrespective of socioeconomic disadvantage or whether they participated in the research study.^50^

Key to implementation was a site visit conducted by two investigators (J.T., H.N). During this visit, we held facilitated implementation planning meetings to actively support implementation. We conducted four highly successful site visits before March 2020. However, the COVID-19 pandemic effectively eliminated the possibility of in-person site visits. In addition, it increased the complexity of the clinical environment and reduced capacity for clinical teams to take on new initiatives. Although it is understandable that the rate of clinics offering the DA was low given the context, it was disappointing and limited our ability to fully assess the impact of the DA. Our findings confirmed that the patients perceived the DA to be helpful. It is possible that our sites would have found that the benefits of the DA on patients’ experiences outweighed the nominal time required to deliver the DA. Future work will explore other factors that influenced DA implementation.

This study focused on the impact that the DA had on patient engagement. Prior studies examining the DAs have focused on alternative outcomes such as knowledge, decisional regret, and decisional conflict.^51^ Although these all are highly relevant for decisions surrounding breast cancer surgery, we were most interested in how the DA changes the way patients participate in decision-making because we hypothesized that low engagement contributes to socioeconomic disparities. We chose two complementary outcomes. The first evaluated patients’ perception of their confidence in interacting with providers (PEPPI-5).^32,33^ The trial participants reported high self-efficacy in interacting with providers, consistent with other studies evaluating surgical decisions.^52^ Although this limited the utility of this measure as a trial outcome, the patients’ high-level of confidence was encouraging, especially given the diverse cohort of the patients enrolled.

We observed more variation in Active Patient Behaviors,^34–36^ which was captured as a direct count of specific patient behaviors, and hence did not have a ceiling effect. This second outcome has been used extensively in prior work within cancer. Although this is a simple outcome at face value, complexities inherent to the clinical environment can influence counts, irrespective of any intervention. For example, we found that in some cases, Active Patient Behaviors correlated with consultation length, with shorter consultations having fewer opportunities for engagement.

The majority of the patient behaviors fell into the Asking Questions subcategory. We operationalized this subcategory quite broadly to include any utterance in interrogative form asking for information. Although some patient questions are more pertinent to breast cancer surgery decisions than others, we decided that any time a patient felt comfortable asking the surgeon a question was relevant. However, this broad definition may have impacted the sensitivity of this outcome to a DA specifically targeting the surgery decision. Assertive Responses may be the behavior most important for assessing patient engagement in decision-making because these are most directly associated with the decision process. The trend we observed toward increased Assertive Responses for patients in the DA arm is an interesting area for future exploration.

An important objective was to understand how a DA impacts engagement for patients who are socioeconomically disadvantaged. Overall, we observed that socioeconomically disadvantaged patients were less engaged in the consultation. However, the DA was not differentially effective for this group. This is an important observation given prior literature suggesting that disadvantaged patients may experience the greatest gains from a DA.^2^ The low uptake of the DA may have contributed to this discrepancy because the patients in our study who were black, were socioeconomically disadvantaged, or had less education were less likely to review the DA even after accepting it. It is also possible that the type of DA (web-based, patient-directed) and the delivery method (electronic delivery before the consultation) may not have been optimal for disadvantaged patients.^13^ That said, the patients who reviewed the DA rated it favorably irrespective of socioeconomic status. Additional study is needed to understand the association between socioeconomic disadvantage and engagement in order to identify adjunct interventions to support these patients.

Our study had limitations. The COVID-19 pandemic had a significant impact on the performance of this stepped-wedge trial. The progression of a stepped-wedge trial is based on time rather than accrual. Critically, a clinical site transitions through the stepped-wedge waves even if no accrual occurs.^53^ During the pandemic, enrollment fluctuated significantly across sites based on external factors, including temporary closures of all Alliance clinical trials, site-specific holds in clinical trial accrual, and competing priorities for clinical teams. This resulted in necessary revisions to the analytic plan to account for time and the relationship between patient enrollment and COVID.

Importantly, even with our reduced sample size, we were adequately powered to address study objectives. Our study findings also suggest that the COVID-19 pandemic had a direct effect of increasing patient engagement during the consultation, irrespective of the DA. In our exploratory analysis, this change appeared to be largely driven by an increase in Asking Questions, although an increase in Assertive Responses also was noted. As described earlier, we counted any question the patient asked. We did not subcategorize the type of question and therefore cannot provide more granular insight into how the pandemic influenced questions.

In addition, the nature of the clinical encounter changed dramatically during the course of this study because of COVID-19, with periods of time in which patients were not allowed to have family support, patients and providers required to wear masks and/or face shields, and clinic changes in flow. Thus, it is not surprising that we noticed an association between the COVID-19 pandemic and Active Patient Behaviors.

Finally, a minority of the patients reviewed the DA. Although the cause of this was likely multi-factorial, the competing priorities facing clinical teams during the pandemic inevitably led to lower implementation. We attempted to address the low use of the DA through the treatment-compliant analysis. The question used to select patients for the treatment-compliant cohort differed between the arms, but this was the best approximation we could make with the available data. It is possible that we underestimated the effect of the DA in our analyses.

Despite all the challenges we faced in the performance of this trial, we successfully enrolled almost 600 patients with diverse characteristics from 10 clinics. Although a minority of the patients reviewed the DA before their consultation, those patients who did review it found the DA helpful and would recommend it to others. We plan to continue examining our data to understand the effect of the DA on those who reviewed it, as well as to understand factors leading to a lower rate of review for minority patients, those socioeconomically disadvantaged, and those with lower education.

## Conclusion

In this trial conducted in clinics that serve diverse populations, no significant relationship was observed between a web-based DA and patient engagement. However, conducting this stepped-wedge trial within the context of the pandemic was challenging. Future analyses will explore the factors associated with implementation of the DA in the clinics, characteristics of patients who reviewed the DA, and persistent barriers to engagement.

## Appendix

See Table 6, 7, 8 and 9.

**Table 6:** Sensitivity analysis for the impact of a web-based decision aid on PEPPI-5 with imputation of missing outcomes (*n* = 63 with imputed outcomes)

Effect	Parameter estimate (95 % CI)	*p* Value
Intervention status: 1 versus 0 (reference)	− 0.6 (− 1.9 to 0.8)	0.367
Post-COVID restart: 1 versus 0 (reference)	− 1.3 (− 2.9 to 0.4)	0.122
Within-surgeon ICC = 0.039		

*PEPPI* Patient’s self-efficacy in patient-physician interactions, *CI* confidence interval, *ICC* intraclass correlation coefficient

**Table 7:** Sensitivity analysis for the impact of a web-based decision aid on active patient behaviors with imputation of missing outcomes (*n* = 9 with imputed outcomes)

Effect	Parameter estimate (95 % CI)	*p* Value
Intervention status: 1 versus 0 (reference)	2.2 (–4.7 to 9.1)	0.507
Post-COVID restart: 1 versus 0 (reference)	9.8 (1.5 to 18.1)	0.024
Within-surgeon ICC = 0.141;		

*PEPPI-5* Patient’s self-efficacy in patient-physician interactions, *CI* confidence interval, *ICC* intraclass correlation coefficient

**Table 8:** Characteristics of patient participants in treatment compliant cohort (*n* = 290)

	Usual care (*n* = 154) *n* (%)	Decision aid (*n* = 136) *n* (%)
Median age: years (range)	58 (47–68)	61 (50–66)
Race White Black Other	103 (67) 31 (20) 20 (13)	99 (73) 16 (12) 21 (15)
Hispanic or Latino	5 (3)	7 (5)
Median ADI decile number (range)	5 (1–10)	4 (1–10)
Socioeconomic disadvantage	37 (24)	15 (11)
T stage T0 T1/T2 T3/T4 Tx or missing	20 (13) 117 (76) 12 (7) 5 (3)	25 (18) 97 (71) 13 (10) 1 (1)
Nodal status N0 N1/N2/N3 Nx or missing	116 (75) 22 (14) 16 (10)	113 (83) 18 (13) 5 (4)
Enrollment post-COVID	50 (33)	127 (93)
*Sites*		
Augusta University Medical Center	10 (7)	6 (4)
Bay Area Breast Surgeons Inc	13 (8)	9 (7)
Billings Clinic Cancer Center	13 (8)	0 (0)
Carle Cancer Center	12 (8)	24 (18)
John H Stroger Jr Hospital of Cook County	6 (4)	6 (4)
Kapiolani Medical Center for Women and Children	18 (12)	18 (13)
Marshfield Medical Center-Marshfield	26 (17)	3 (2)
Medical University of South Carolina	18 (12)	22 (16)
University of New Mexico Cancer Center	4 (3)	27 (20)
Virginia Commonwealth University/Massey Cancer Center	34 (22)	21 (15)

*ADI* Area deprivation index

**Table 9:** Treatment-compliant analysis for impact of a web-based decision aid on engagement

Effect	Parameter estimate (95 % CI)	*p* Value
**A4a** Effect for the impact of a web-based decision aid on PEPPI-5^a^ (*n* = 271)		
Decision aid versus usual care (reference)	− 0.9 (− 2.4 to 0.7)	0.267
Enrollment post-COVID: 1 versus 0 (reference)	− 2.3 (− 4.1 to − 0.5)	0.014
Within-surgeon ICC = 0.0079		

**A4b** Effect for the impact of a web-based decision aid on active patient behaviors^a^ (*n*=285)		
Decision aid versus usual care (reference)	5.4 (− 4.4 to 15.1)	0.278
Enrollment post-COVID: 1 versus 0 (reference)	11.9 (0.6 to 23.2)	0.039
Within-surgeon ICC = 0.0407 and within-site-between surgeon ICC = 0.1067		

*PEPPI-5* Patient’s self-efficacy in patient-physician interactions, *ICC* intraclass correlation coefficient, *CI* confidence interval

^a^Models adjusted for wave, wave-squared, and post-COVID restart time
